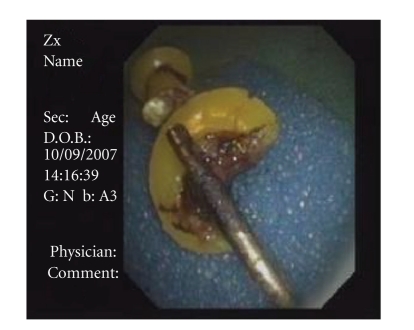# Laser-Assisted Removal of Aspirated Thumbtacks by Flexible Bronchoscopy

**DOI:** 10.1155/2010/598760

**Published:** 2010-06-14

**Authors:** Oren Fruchter, Benjamin D. Fox, Yael Raviv, Mordechai R. Kramer

**Affiliations:** The Pulmonary Institute, Rabin Medical Center, Beilinson Campus, Petah Tiqwa 49100, Israel

## Abstract

*Background*. Aspirated thumbtacks are difficult to extract as the sharp edge of the thumbtack often is well imbedded within bronchial wall and its removal is technically demanding and may cause complications such as bronchial mucosal tear and bronchial wall perforation. These sharp metal objects are commonly removed using rigid bronchoscopy since their removal through flexible bronchoscopy is considered to be dangerous. *Objectives*. To describe a technique for removal of sharp aspirated metal objects employing laser through flexible bronchoscopy. *Methods*. We report two patients in whom a new technique for removal of sharp aspirated metal objects utilizing Nd-Yag laser flexible bronchoscopy was used. *Results*. Successful and uncomplicated removal of the aspirated thumbpack by flexible bronchoscopy under conscious sedation was accomplished in the two patients described. Both patients were discharged within 24 hours. *Conclusions*. In patients with aspirated thumbtack laser-assisted breakage of the object through flexible bronchoscopy may obviate the need for rigid bronchoscopy or thoracotomy.

## 1. Introduction

Aspirated tracheobronchial foreign bodies (FB) are often difficult to diagnose as the cause of obstructive pneumonias and atelectasis, but once discovered, they can generally be removed by endoscopy, leading to immediate and permanent resolution of symptoms [1]. The first priority is to remove the FB using either rigid or flexible bronchoscope. Aspirated thumbtacks are difficult to extract as the sharp edge of the thumbtack often is well imbedded within bronchial wall and its removal is technically demanding and may cause complications such as bronchial mucosal tear and bronchial wall perforation [2]. These sharp metal objects are commonly removed using rigid bronchoscopy since their removal through flexible bronchoscopy is considered to be dangerous. 

 We describe two patients in whom a technique for removal of sharp aspirated metal objects utilizing Nd-Yag laser flexible bronchoscopy was used.

## 2. Case Reports

**Case 1**

**Figure 1:**
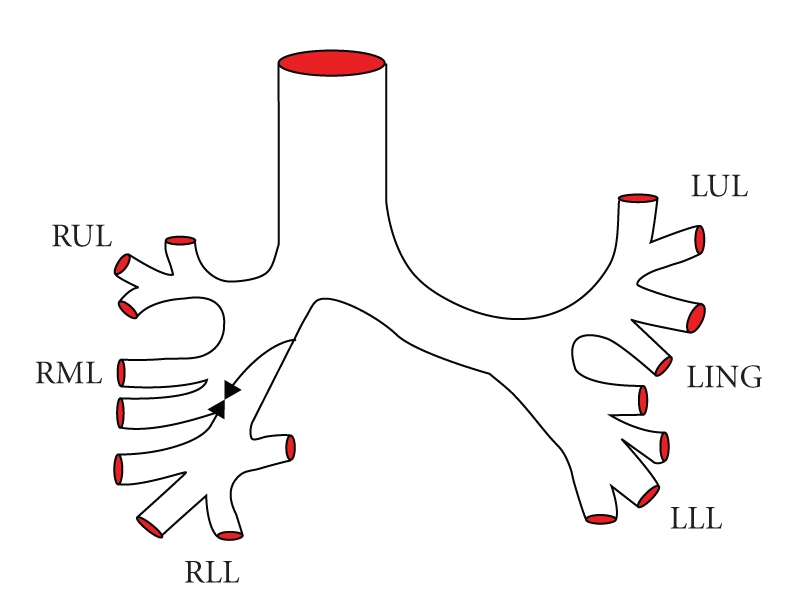


**Figure 2:**
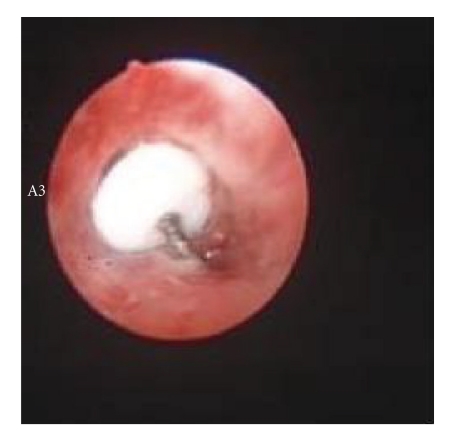


**Figure 3:**
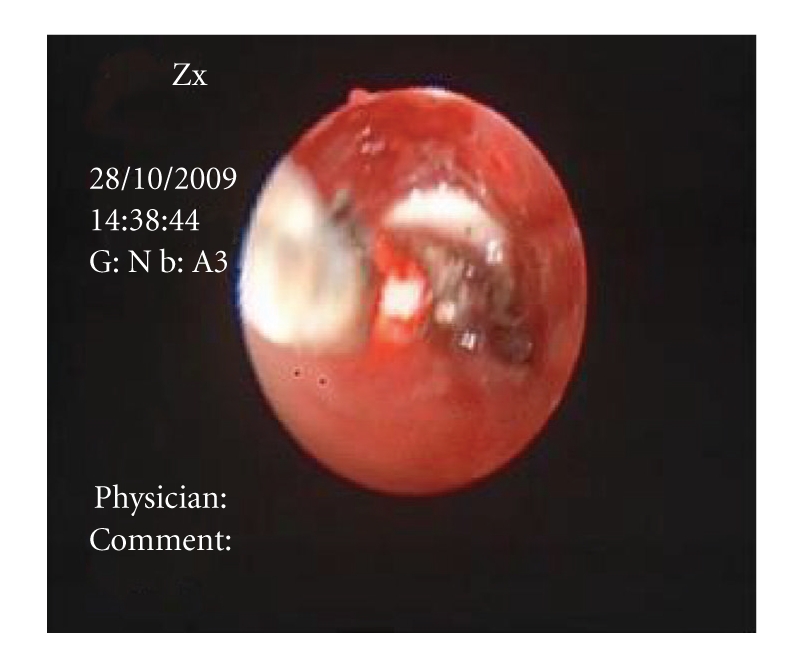


A previously healthy 25-year-old female presented with difficulty in breathing and localized wheezing four hours following accidental aspiration of a thumbtack during repair works. Chest X-ray revealed aspirated thumbtack positioned in the bronchus intermedius. FOB was performed by an experienced pulmonologist using a videobronchoscope (Olympus BF-P240, Olympus, Tokyo, Japan) under sedation and local anesthesia. Monitoring during bronchoscopy included pulse oximetry, ECG, and BP measurements. Flexible bronchoscopy revealed a thumbtack deeply wedged in the mid bronchus intermedius with its sharp edge deeply embedded within the bronchial mucosa (Figures 1 and 2). Several attempts to retrieve the thumbtack by forceps were unsuccessful as the needle went further into the bronchial wall upon repeated manipulations. Nd:YAG Laser (SHARPLAN −3000, SHRPLAN Lasers) was used to cut and break the plastic base of the thumbtack employing 11920 Joules (Figure 3). Following this maneuver, the sharp spike and the broken plastic base were easily removed from the bronchial wall using standard forceps (Olympus FB 20 C). The patient had an uneventful recovery.

**Case 2**

A healthy 41-year-old male presented several hours following accidental aspiration of a thumbtack that occurred during house works. Flexible bronchoscopy performed in another hospital revealed a thumbtack deeply wedged in the proximal bronchus intermedius with its sharp edge deeply embedded within the bronchial mucosa. Multiple attempts to remove the thumbtacks in another hospital both by flexible and rigid bronchoscopy were unsuccessful as the sharp end of the thumbtacks went into the bronchial wall and surgery was considered. The patient was referred to our institute and Nd:YAG Laser was used to cut and break the plastic base of the thumbtack employing 8700 Joules. This was followed by removal of the separated plastic base and sharp spike of the thumbtack using biopsy forceps (Figure 4).

## 3. Discussion

FB aspiration remains a common problem especially among young children but also in adults. In adults, most of FB aspirations are seen in the 6th or 7th decade of life when airway protective mechanisms function inadequately, for example, due to central nervous system dysfunction, intubation or facial traumas [1]. Thumbtack aspiration usually occurs during repair works or house decorating activities during which the patient holds the thumbtack between their lips while using their two hands to hold an object. Any maneuver, such as laughter, talking, and coughing then predispose them to aspiration. Aspiration of such a sharp metal foreign body can be a life-threatening event in itself if chocking occurs. In addition iatrogenic complications during its removal are frequently encountered and include laceration of the bronchial mucosa and airway perforation leading to bleeding and mediastinitis [2]. In contrast to other forms of FB aspiration, thumbtack aspirations tend to be easily diagnosed as all of these inhaled FBs are radio-opaque and, as such, can be picked up easily by chest radiography. Once diagnosed by means of radiography, these inhaled pins should usually be removed by rigid bronchoscopy. However, rigid bronchoscopy requires general anesthesia and operating room setting. In addition, in one of the described cases retrieval efforts by rigid bronchoscopy were unsuccessful in removing the thumbtack. The main difficulty in removing a thumbtack is that its sharp edge tends to be deeply lodged within the bronchial mucosa and the limited space within the bronchial lumen makes its safe removal that is usually carried out by a forceps, difficult and dangerous. The literature on the use of laser to facilitate FB removal from the tracheobronchial tree is limited. Boelcskei et al. [3], reported on a 7-year-old patient in whom laser was used to vaporize a granulation tissue that was covering an aspirated nut shell that was positioned in the left mainstem bronchus. Hayashi et al. [4] reported on a 19-month old patient in whom laser was used to cut an aspirated chicken bone that was subsequently removed by a regular forceps. Our case series is unique in several aspects. This is the first case of laser assisted removal of a nonorganic aspirated metal object. We did not use a rigid bronchoscope and the entire procedure was carried out through a flexible bronchoscope under conscious sedation. Important technical notes are that it requires use of high power laser (around 1000 Joules), one should keep in mind to avoid intrabronchial ignition and fire by keeping the concentration of inspired oxygen concentration to as low as possible (less than 40%)—.In addition it is important to keep the laser fiber remote enough from the plastic base of the thumbtack in order to avoid it from sticking to the tip of the fiber during its melting. However, one alternative maneuver that should be explored is by sticking the melted FB to the laser fiber and pulling it along with the fiber and the bronchoscope. The major limitations of removing a thumbtack using flexible bronchoscope is that it could be dislodged from the forceps and be wedged into a distal inaccessible position in the trache-bronchial tree or alternatively causing mucosal tear. 

In conclusion, in patients with aspirated thumbtack laser-assisted breakage of the object may obviate the need for thoracotomy.

## Figures and Tables

**Figure 4:**